# Efficacy and Safety of Corticosteroids in Tuberculous Meningitis: Systematic Review, Meta‐Analysis, and Meta‐Regression

**DOI:** 10.1002/brb3.71639

**Published:** 2026-07-28

**Authors:** Ayesha Younas, Rizwana Noor, Muhammad Talha Shaukat, Aisha Ijlal, Irra Tariq, Murtaja Satea, Muhammad Sameer Almas, Rosheen Jamil, Mifrah Rahat Khan Sherwani, Norma Nicole Gamarra Valverde, Maria Qadri, Mahwash Siddiqi, Kashif Qureshi, Kivanc Yangi, Bipin Chaurasia

**Affiliations:** ^1^ Department of Medicine Allama Iqbal Medical College Lahore Pakistan; ^2^ Department of Medicine Khyber Medical College Peshawar Pakistan; ^3^ Department of Medicine King Edward Medical University Lahore Pakistan; ^4^ Department of Physical Therapy Jinnah Sindh Medical University Karachi Pakistan; ^5^ Department of Medicine United Medical and Dental College Karachi Pakistan; ^6^ Department of Surgery College of Medicine University of Warith Al‐Anbiya Karbala Iraq; ^7^ Department of Medicine Jefferson Abington Memorial Hospital Pennsylvania USA; ^8^ Department of Cardiology Duke University Health Durham North Carolina USA; ^9^ Department of Medicine Universidad Peruana Cayetano Heredia Lima Peru; ^10^ Department of Medicine Jinnah Sindh Medical University Karachi Pakistan; ^11^ Department of Medicine Penn State Health Milton S. Hershey Medical Centre Hershey Pennsylvania USA; ^12^ Department of Neurosurgery Yale School of Medicine New Haven Connecticut USA; ^13^ Department of Neurosurgery Barrow Neurological Institute Phoenix Arizona USA; ^14^ Department of Neurosurgery Neurosurgery Clinic Birgunj Nepal

**Keywords:** Adrenal cortex hormones, dexamethasone, glucocorticoids, meta‐analysis, systematic review, tuberculous meningitis

## Abstract

**Background:**

Tuberculous meningitis (TBM) carries a case fatality rate of up to 50% in resource‐limited settings despite standard anti‐tuberculosis therapy. Adjunctive corticosteroids are widely recommended, yet their effect on mortality and safety across diverse populations remains imprecisely defined.

**Methods:**

We conducted a systematic review and meta‐analysis of randomized and quasi‐randomized controlled trials identified through PubMed, Cochrane Library, ClinicalTrials.gov, Scopus, and Google Scholar up to May 2025. The outcomes were all‐cause mortality, gastrointestinal bleeding, visual and auditory impairment, hydrocephalus, and joint disorders. Statistical heterogeneity was assessed using the I^2^ statistic. Certainty of evidence was assessed with the GRADE framework.

**Results:**

Eleven studies enrolling 1917 patients were included. Corticosteroids significantly reduced all‐cause mortality (RR = 0.84, 95% CI: 0.76–0.94, *p* = 0.002) with low heterogeneity (I^2^ = 0%). Subgroup analysis revealed pronounced benefits in low‐income countries (RR = 0.82, CI: 0.72–0.95) and dexamethasone use (RR = 0.87, CI: 0.77–0.97). In HIV‐positive patients, corticosteroids did not significantly impact mortality (RR = 0.90, CI: 0.77–1.05, *p* = 0.18). No statistically significant increase in adverse outcomes was detected, including gastrointestinal bleeding (RR  =  0.87), visual impairment (RR  =  1.62), auditory impairment (RR  =  0.79), hydrocephalus (RR  =  0.47), or joint disorders (RR  =  0.63).

**Conclusion:**

Adjunctive corticosteroids, particularly dexamethasone, significantly reduce mortality in TBM without increasing major adverse effects. The absence of benefit in HIV‐positive patients highlights the need for targeted trials in this subgroup and in pediatric populations.

## Graphical abstract

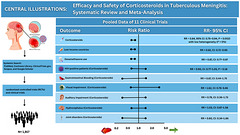

## 1 Introduction

The most common and most lethal neurological manifestation of Mycobacterium tuberculosis (MTB) is tuberculous meningitis (TBM) (Wang et al. 2024). It constitutes approximately 5%–10% of extrapulmonary tuberculosis cases and around 1% of all active tuberculosis cases (Wang et al. 2019). Following inhalation of airborne bacilli and primary pulmonary infection, hamatogenous dissemination can seed the central nervous system, resulting in TBM (Oo and Agrawal 2025).

In TBM, bacilli infiltrate the meninges and cerebrospinal fluid (CSF). Children below four years of age, the aging population, and people with HIV are at high risk of getting infected (Dodd et al. 2021; Wilkinson et al. 2017). Despite prompt treatment, the probability of long‐term neurological morbidity and mortality is high (Wang et al. 2024). TBM is a primary health issue of concern on the global level, with Southeast Asia and Africa accounting for approximately 44% and 25% of the global tuberculosis burden, respectively (Leiva‐Ordoñez and Quintero 2026). The average case fatality rate is estimated at around 27% even with standard anti‐tuberculosis therapy and may exceed 50% in resource‐limited settings (Dodd et al. 2021; Ieque et al. 2025).

The World Health Organization's Global Tuberculosis Report 2025 states that TB killed 1.23 million people in 2024, including 150,000 HIV‐positive individuals, solidifying its ranking as the leading infectious disease‐related cause of death globally (World Health Organization, n.d.).

The outcome of TBM is heavily influenced by the intensity of intracerebral inflammation. Corticosteroids have long been proposed to mitigate this inflammatory response and improve survival outcomes. Although the concept emerged in the 1950s, it took decades of accumulated clinical evidence for corticosteroids to be widely recommended in treatment protocols (Prasad et al. 2016; Thwaites et al. 2004). Several international and national guidelines, including those from the American Thoracic Society and British Infection Society, endorse adjunctive corticosteroids in the management of TBM (Nahid et al. 2016; Thwaites et al. 2009).

However, uncertainties persist regarding their true clinical benefit. Specifically, the extent of corticosteroid impact on mortality, long‐term neurological sequelae, and treatment‐related adverse events remains unclear. Previous meta‐analyses and systematic reviews have produced conflicting findings, limited by small sample sizes, heterogeneous study designs, and the absence of advanced analytical methods such as meta‐regression.

The clinical question guiding this review was structured using the PICO framework: Population: adults and children diagnosed with tuberculous meningitis (TBM) of any severity grade and HIV status; Intervention: adjunctive corticosteroid therapy (dexamethasone, prednisolone, prednisone, methylprednisolone, or hydrocortisone) in addition to standard anti‐tuberculosis treatment; Comparator: placebo or standard anti‐tuberculosis treatment alone; Outcomes: all‐cause mortality (primary); gastrointestinal bleeding; visual impairment; auditory impairment; hydrocephalus; and joint disorders (secondary). This review builds upon prior syntheses, including the 2016 Cochrane review by Prasad et al., 2016 in several key respects. First, it incorporates contemporary RCTs published up to May 2025, including the landmark Donovan et al. (2023) trial in HIV‐positive patients, which substantially alters the evidence base for this population. Second, it extends prior work by conducting formal meta‐regression to assess whether follow‐up duration and year of publication moderate the mortality effect, and by applying GRADE certainty‐of‐evidence assessments across all outcomes. Third, the subgroup analysis stratified by World Bank income classification and corticosteroid type provides actionable, policy‐relevant estimates not previously reported. Therefore, we conducted this systematic review, meta‐analysis, and meta‐regression to provide the most current and methodologically rigorous synthesis of randomized evidence on adjunctive corticosteroid therapy in TBM. Specifically, we sought to (i) quantify the effect of corticosteroids on all‐cause mortality among patients with confirmed TBM; (ii) evaluate their impact on key neurological and systemic adverse outcomes, including visual impairment, auditory deficits, hydrocephalus, gastrointestinal bleeding, and joint disorders; and (iii) investigate potential sources of heterogeneity by assessing the modifying roles of HIV status, steroid type, geographical and economic setting, and follow‐up duration through subgroup analyses and meta‐regression.

## 2 Methods

This protocol for a systematic review was prospectively registered under registration number CRD42024541606 with the International Prospective Register of Systematic Reviews (PROSPERO). The Cochrane Handbook of Intervention (Higgins et al. 2011) standards were followed in conducting this systematic review and meta‐analysis, and the Preferred Reporting Items for Systematic Reviews and Meta‐Analysis (PRISMA) recommendations were followed in reporting.

### 2.1 Literature Search

A comprehensive literature search of major bibliographic databases (PubMed, Cochrane, clinicaltrials.gov, Scopus, and Google Scholar), each searched from inception to May 2025, was conducted for randomized controlled trials (RCTs), including older trials that used quasi‐random or unclearly reported allocation. Hand searching was also done to identify any relevant articles not retrieved otherwise. The search strategy combined two concept blocks: (1) disease terms, including the MeSH heading Tuberculous Meningitis and free‐text synonyms (tuberculous meningitis, TB meningitis, neurotuberculosis, CNS tuberculosis, tuberculous meningoencephalitis, and mycobacterial meningitis); and (2) intervention terms, including the MeSH heading Adrenal Cortex Hormones and free‐text synonyms (corticosteroids, glucocorticoids, dexamethasone, prednisolone, prednisone, methylprednisolone, hydrocortisone, betamethasone, triamcinolone, and fludrocortisone). The two concept blocks were combined using the Boolean AND operator. Eligibility was restricted to studies available as full text in English; this English‐language restriction is acknowledged as a potential limitation (Section 4.5). Full search strings for each individual database are provided in the Supplementary Material. For Google Scholar, records were sorted by relevance and screened against the eligibility criteria until successive result pages yielded no further eligible records. The search strategy included all relevant keywords and PubMed entry terms for “Meningeal Tuberculosis” and “Corticosteroids” searched in different combinations to retrieve desired results. The previous meta‐analyses were also searched. For inaccessible articles, an attempt to contact the author was made via electronic mail; however, to no avail. Snowball citation searching was used to supplement the database search. We applied both backward citation tracking by reviewing the reference lists of included studies and forward citation tracking by using tools such as Google Scholar and Scopus to identify more recent studies that cited the included articles. This method helped identify additional relevant studies that may not have appeared in the initial keyword‐based database searches to include all trials from inception till May 2025. Relevant search strategies for individual databases are recorded in the Supplementary Material.

### 2.2 Study Selection Criteria

**FIGURE 1:**
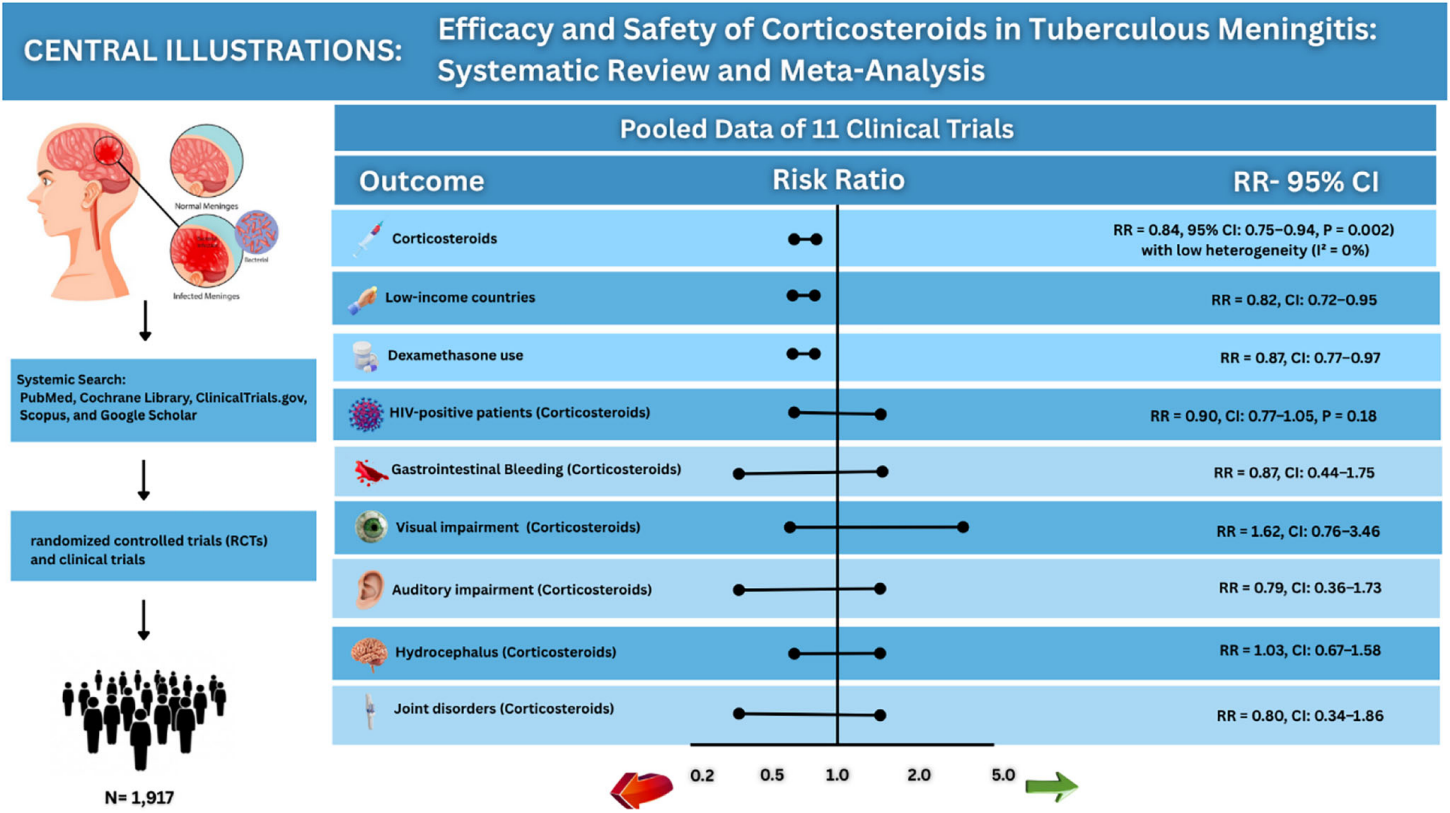
Central illustration: Summary of efficacy and safety outcomes of corticosteroids in TB meningitis.

**FIGURE 2:**
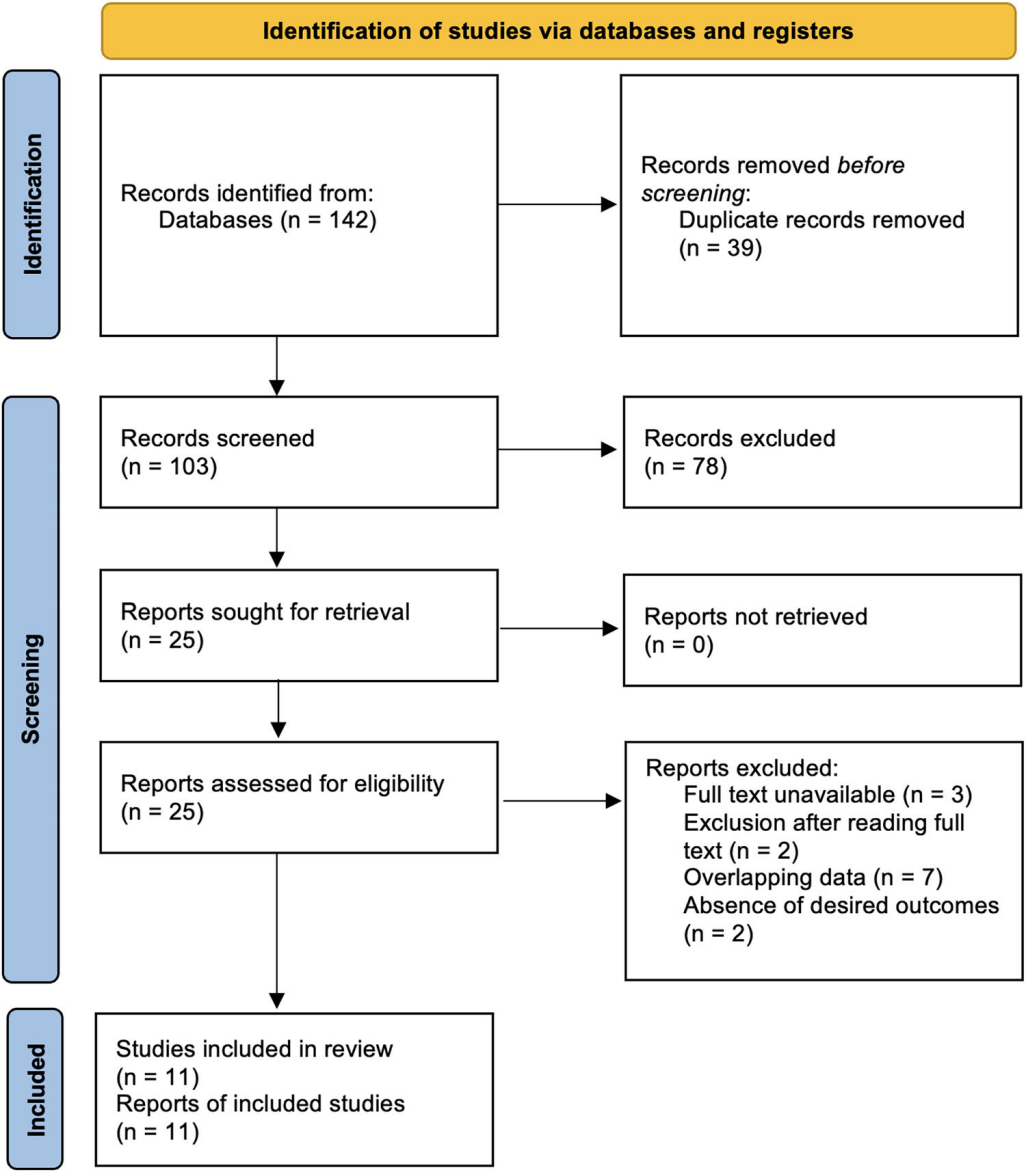
Prisma flowchart for the study selection process.

All the retrieved studies from databases were imported to Rayyan.ai, and 39 duplicates were resolved. Three authors independently initiated screening with the blind on. Upon primary screening, authors included trials with relevant keywords in the title of the study. After resolving conflicted studies, the total number of articles yielded on primary screening was 25. The blind was turned on again, and secondary screening was started where full‐length articles were read. Sixteen articles were excluded based on unavailability of full text, absence of desired outcomes, overlapping results, and failure to meet the inclusion criteria. On citation and hand searching, two further studies were retrieved, making the final number of studies equal to 11 (Chotmongkol et al. 1996) (Figures 1 and 2; Diao et al. 2020; Donovan et al. 2023; Escobar et al. 1975; Ghosh et al. 1971; Girgis et al. 1991; Higgins et al. 2011; Kalita and Misra 2001; Kumarvelu et al. 1994; O'Toole et al. 1969; Schoeman et al. 1997; Thwaites et al. 2004). In case of any disagreement in the study selection procedure, a consensus was reached to ensure objectivity and fairness.

### 2.3 Eligibility Criteria

#### 2.3.1 Inclusion Criteria

Studies were included if they met all the following conditions: (i) Randomized controlled trials, including quasi‐randomized trials, with either parallel or factorial designs. (ii) Human participants of any age or sex diagnosed with TBM, confirmed either by clinical criteria, CSF analysis, imaging, or microbiological evidence of MTB. (iii) Administration of corticosteroids (including dexamethasone, prednisolone, prednisone, methylprednisolone, or other glucocorticoids) as adjunctive therapy to standard anti‐tuberculosis treatment, regardless of the dose, route (oral, intravenous, or intramuscular), or duration. (iv) Placebo or standard anti‐tuberculosis regimen without corticosteroid therapy. (v) Studies that reported at least one of the following outcomes: all‐cause mortality, neurological sequelae, gastrointestinal bleeding, hydrocephalus, visual or auditory impairment, or joint disorders. (vi) Full‐text articles published in English.

#### 2.3.2 Exclusion Criteria

The following studies were excluded: (i) Non‐interventional studies such as case reports, observational studies, cohort studies, cross‐sectional studies, narrative reviews, systematic reviews, conference abstracts, posters, and letters to the editor. (ii) Unpublished or ongoing clinical trials without available outcome data. (iii) Closed‐access or inaccessible articles where full text could not be retrieved even after attempts to contact the authors. (iv) Animal or in vitro studies evaluating corticosteroid effects on TBM models. (v) Studies involving other forms of meningitis (bacterial, viral, or fungal) or mixed populations where data for TBM could not be separately extracted. (vi) Duplicate publications or overlapping datasets reporting the same patient cohort without additional unique data.

Only randomized and quasi‐randomized controlled trials were eligible; non‐randomized observational designs (cohort, case‐control, cross‐sectional, and case series) were excluded per the criteria above. Because the number of RCTs of adjunctive corticosteroids in TBM is limited and several foundational trials predate modern randomization standards, older quasi‐randomized trials were retained to maximize the evidence base, and their method of randomization was appraised within the risk‐of‐bias evaluation (Section 2.5). These criteria ensured the inclusion of controlled clinical evidence focused exclusively on human randomized and quasi‐randomized evaluations of corticosteroid therapy in TBM.

### 2.4 Data Extraction

All the pertinent information was extracted by two authors (S.A. and I.T.) using a standardized data extraction form in all the studies that were included, and any differences that were exhibited by these authors were settled by reaching a consensus with one more author. The information gathered included year of publication, first author, country, study design, and overall sample size, including division into groups that were treated with corticosteroids and control groups. The participants data included mean or median age with standard deviation or interquartile range, HIV status, and baseline TBM grade. Intervention details such as corticosteroid type (e.g., dexamethasone, prednisolone, and methylprednisolone), dose, route (oral, intravenous, or intramuscular), and duration of treatment were also retrieved with the nature of the comparator group, which included either a placebo or standard anti‐tuberculosis treatment. The time and frequency of outcome assessments were also recorded.

To perform quantitative synthesis, the number of events and the total number of participants in each group were read in the case of dichotomous outcomes (all‐cause mortality, gastrointestinal bleeding, hydrocephalus, visual impairment, auditory impairment, and joint disorders), and mean ± SD numbers were read in the cases of continuous outcomes. All main and secondary outcomes included reported *p*‐values, reported confidence intervals, and reported effect estimates (risk ratios, odds ratios, or hazard ratios). All data were cross‐verified to check completeness and accuracy of such data before analysis, and where missing or unclear information was needed, efforts were made to have clarification with the respective study author.

### 2.5 Risk of Bias

Two authors (M. T. S. and R. N.) independently conducted the quality assessment. Any discrepancy regarding the risk of bias assessment was settled by consensus. The risk of bias of the included trials was assessed using the revised Cochrane risk‐of‐bias tool for randomized trials (RoB 2) (Sterne et al. 2019), with each study rated as low risk, some concerns, or high risk. Assessment covered the five RoB 2 domains: bias arising from the randomization process, bias due to deviations from intended interventions, bias due to missing outcome data, bias in measurement of the outcome, and bias in selection of the reported result, together with an overall risk‐of‐bias judgment. For older trials that used quasi‐random or unclearly described allocation, this was captured within the randomization‐process domain. Any disagreements were settled by the third author (A.Y). (Figure 2).

### 2.6 Statistical Analysis

Review Manager (RevMan) version 5.4.1 was used for descriptive and quantitative analyses in compliance with the PRISMA 2020 recommendations and the Cochrane Handbook for Systematic Reviews of Interventions. A random‐effects model (DerSimonian and Laird approach) with inverse‐variance weighting was used to assess the results to account for the anticipated variability between studies. The summaries were presented as pooled risk ratios (RRs) together with the associated 95% CIs. The Higgins *I* ^2^ statistic was used to quantify statistical heterogeneity, which was classified as low (≤ 25%), moderate (≈ 50%), or large (≥ 75%). The statistical significance threshold was chosen at *p* < 0.05, and analyses were carried out on an intention‐to‐treat basis.

Subgroup analyses were conducted to examine potential modifiers of effect, including type of corticosteroid (dexamethasone vs. other steroids), HIV status of participants, and income classification of the study setting. The World Bank Income Classification was applied to categorize studies as originating from upper‐middle‐ or lower‐middle‐income countries (Table 1). Publication bias was assessed visually by funnel‐plot inspection and statistically by Egger's regression test using Comprehensive Meta‐Analysis (CMA) software. Meta‐regression analyses were conducted in R version 4.4.0 using the meta and metafor packages to explore the influence of moderators such as follow‐up duration and year of publication. Sensitivity analyses were performed via a leave‐one‐out approach, sequentially omitting high‐risk studies (Escobar 1975; Ghosh 1971; and Kumarvelu 1994) and trials published before 2000 to assess the stability of pooled results.

**TABLE 1:** Baseline clinical characteristics of included articles.

**Study**	**Year**	**Country**	**Income (World Bank)**	**N (Steroid / Control)**	**Design**	**Age**	**Follow‐up**	**Steroid Type**	**Route**	**HIV Status**	**TBM Grade**	***p*‐value**
Thwaites (Donovan et al. 2023)	2004	Vietnam	LMIC	274/271	RCT	43.8 ± 12.9/42.3 ± 12.2 year	9 month	Dexamethasone	IV then oral	Mixed	I, II, III	0.01
Donovan (Kalita and Misra 2001)	2023	Vietnam, Indonesia	LMIC / UMIC	263/257	RCT	35.3 ± 9.0/36.0 ± 9.0 year	12 month	Dexamethasone	IV then oral	HIV+	I, II, III	0.22
Kalita (Escobar et al. 1975)	2001	India	LMIC	21/16	CT	27.2/30.1 year	3 month	Methylprednisolone + prednisolone taper	IV then oral	NS	I, II, III	< 0.01
Escobar (Schoeman et al. 1997)	1975	Colombia	UMIC	52/47	CT	1 mo – 14 year	30 days	Prednisone	Oral	NS	I, II, III	< 0.05
Schoeman (Girgis et al. 1991)	1997	South Africa	UMIC	70/71	RCT	38.3 ± 28.3/28.8 ± 21.9 month	6 month	Prednisone	Oral	NS	II, III	0.015
Girgis (Kumarvelu et al. 1994)	1991	Egypt	LMIC	145/135	CT	5 mo—55 year (median 8 year)	2 year	Dexamethasone	IM	NS	NS	<0.05
Kumarvelu (Ghosh et al. 1971)	1994	India	LMIC	24/23	RCT	26.9 year (mean)	13 month	Dexamethasone	IV then oral	NS	Mild, moderate, severe	NS
Ghosh (O'Toole et al. 1969)	1971	India	LMIC	62/36	CT	<12 year	10 month	Hydrocortisone + prednisolone	Oral + intrathecal	NS	I, II, III	NS
O'Toole (Diao et al. 2020)	1969	India	LMIC	11/12	CT	<2 – >45 year	13 month	Dexamethasone	IV	NS	Moderate, severe	0.70
Chotmongkol (Wang et al. 2022)	1996	Thailand	UMIC	29/30	CT	42.0 ± 18.6/39.0 ± 18.3 year	30 month	Prednisolone	Oral	HIV–	I, II, III	0.25
Diao (Chotmongkol et al. 1996)	2020	China	UMIC	34/34	RCT	45.4 ± 13.7/45.9 ± 13.7 year	6 month	Glucocorticoids	IV then oral	NS	NS	0.046

Age reported as mean ± SD (steroid / control) unless otherwise specified. Schoeman 1997 age reported in months (mean ± SD). *p*‐value corresponds to primary outcome (all‐cause mortality) as reported by study authors.

Abbreviations: CT = controlled trial with quasi‐random or unclear allocation, IM = intramuscular, IV = intravenous, LMIC = lower‐middle‐income country, mo = months, NS = not stated/reported, RCT = randomized controlled trial, UMIC = upper‐middle‐income country, yr = years.

A comprehensive description of the statistical workflow including full search strategies, the PRISMA checklist, meta‐regression model specifications, funnel plots, and sensitivity analyses, is provided in the Supplementary Material (“Supplementary Figures S1–S4” and PRISMA Checklist, Items 5–27).

### 2.7 Certainty of Evidence Assessment

Certainty of evidence was assessed by two authors (M.T.S. and R.N.) according to five grades of Recommendation, Assessment, Development, and Evaluation (GRADE) considerations: risk of bias, inconsistency, indirectness, imprecision, and publication bias. Each body of evidence was rated as being of high, moderate, low, or very low certainty.

## 3 Results

### 3.1 Characteristics of Included Studies: (Figure 1)

This meta‐analysis included 11 eligible randomized and quasi‐randomized controlled trials that involved 1917 patients with tuberculous meningitis (TBM). Out of them, 985 individuals were given adjunctive corticosteroid therapy, and 932 were given conventional anti‐tuberculosis treatment. Two were randomized with a total of 618 subjects, which particularly included HIV‐positive subjects, and the rest of the trials had either negative HIV groups or mixed‐status groups. The median or mean age of the study participants varied considerably, ranging from infancy to over 45 years, since both pediatric and adult populations were included in the studies.

Dexamethasone was the most frequently evaluated corticosteroid, assessed in five trials (Donovan et al. 2023; Girgis et al. 1991; Kumarvelu et al. 1994; O'Toole et al. 1969; Thwaites et al. 2004), and the rest of the studies were on prednisolone, methylprednisolone, or hydrocortisone. In the majority of studies, the severity of the disease was divided into the British Medical Research Council (BMRC) system of grades (Grades I‐III) of mild, moderate, and severe disease manifestations. Corticosteroid administration routes differed across trials, and the most common methods of administration included intravenous or oral routes, which were usually followed by a tapering course.

Baseline demographic information in all studies had equal sexes, mixed samples sizes (between 23 and 531 participants per trial), and mixed geographical distribution in both upper‐middle‐ and lower‐middle‐income nations, such as Vietnam, India, Egypt, Thailand, South Africa, and China. The time used in follow‐up was from a month to 2 years. Table 1 gives a detailed account of baseline demographics and disease characteristics, corticosteroid regimens, and study designs.

### 3.2 Risk of Bias of Included Studies

Methodological quality of the included trials was assessed using the RoB 2 tool. A total of three studies (Escobar et al. 1975; Ghosh et al. 1971; Kumarvelu et al. 1994) were classified as high risk of bias, and this was mainly because there was no clearly defined randomization sequence, allocation concealment was not done, there was no complete outcome data, and there was selective reporting of results. Such studies also showed poor blinding of participants and outcome assessors and this could have led to performance and detection bias. Three more studies (Chotmongkol et al. 1996; Girgis et al. 1991; Kalita and Misra 2001) were reviewed as having some concerns of bias, mostly associated with a lack of sufficient detail about the randomization processes and incomplete description of blinding and outcome measurement.

**FIGURE 3:**
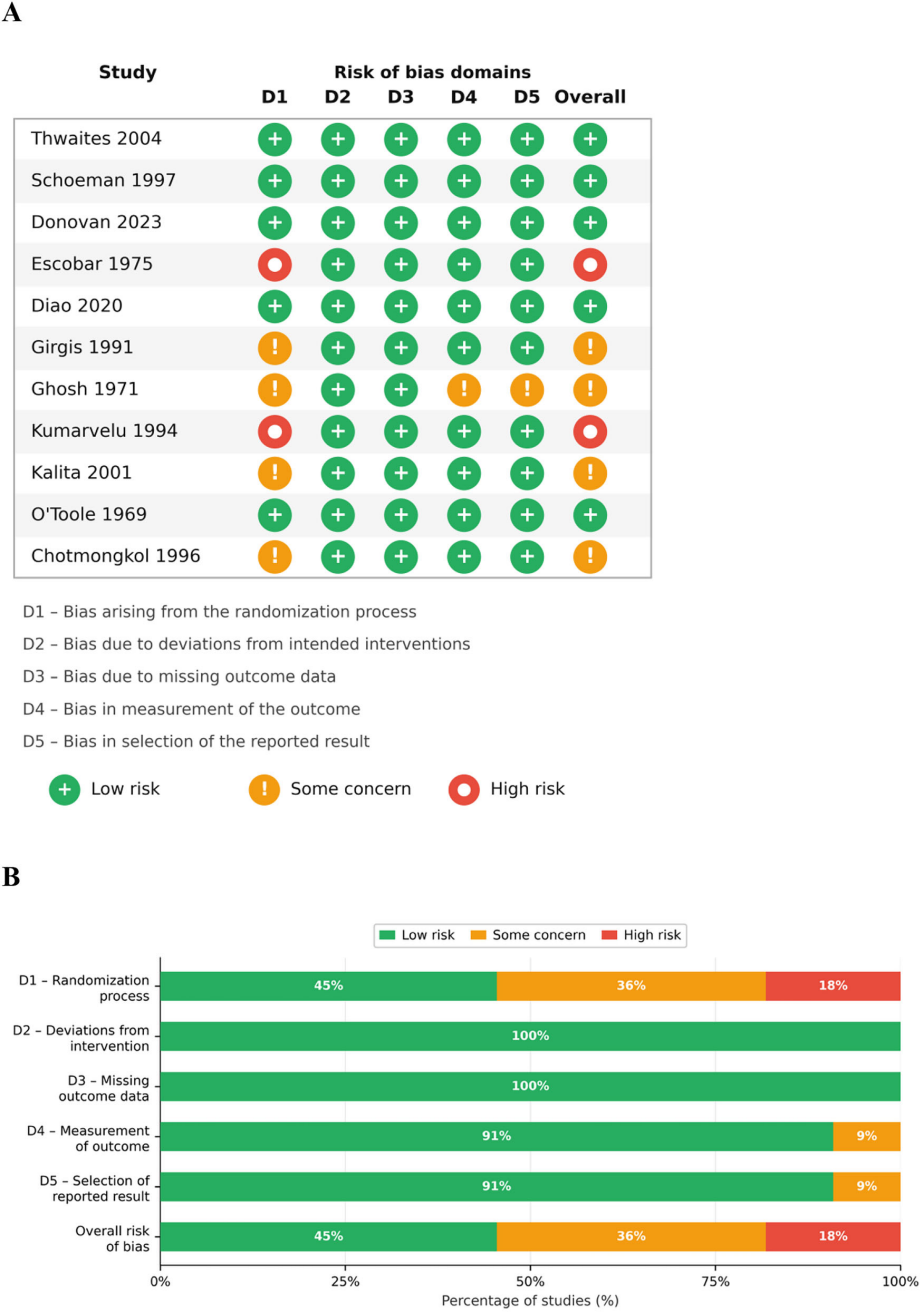
Quality assessment of included trials.

These limitations notwithstanding, the majority of trials described the flow of participants and adherence to the intervention sufficiently and reduced the bias in attrition. The other studies were regarded as being of low risk in all the major areas, such as randomization, allocation concealment, blinding, outcome reporting, and missing data. Overall, the included trials were rated as being at moderate risk of bias, with several older trials rated at high risk in the randomization‐process domain owing to quasi‐random or unclearly reported allocation. Figure 3 gives a graphical summary of the bias distribution in all domains.

**FIGURE 4a:**
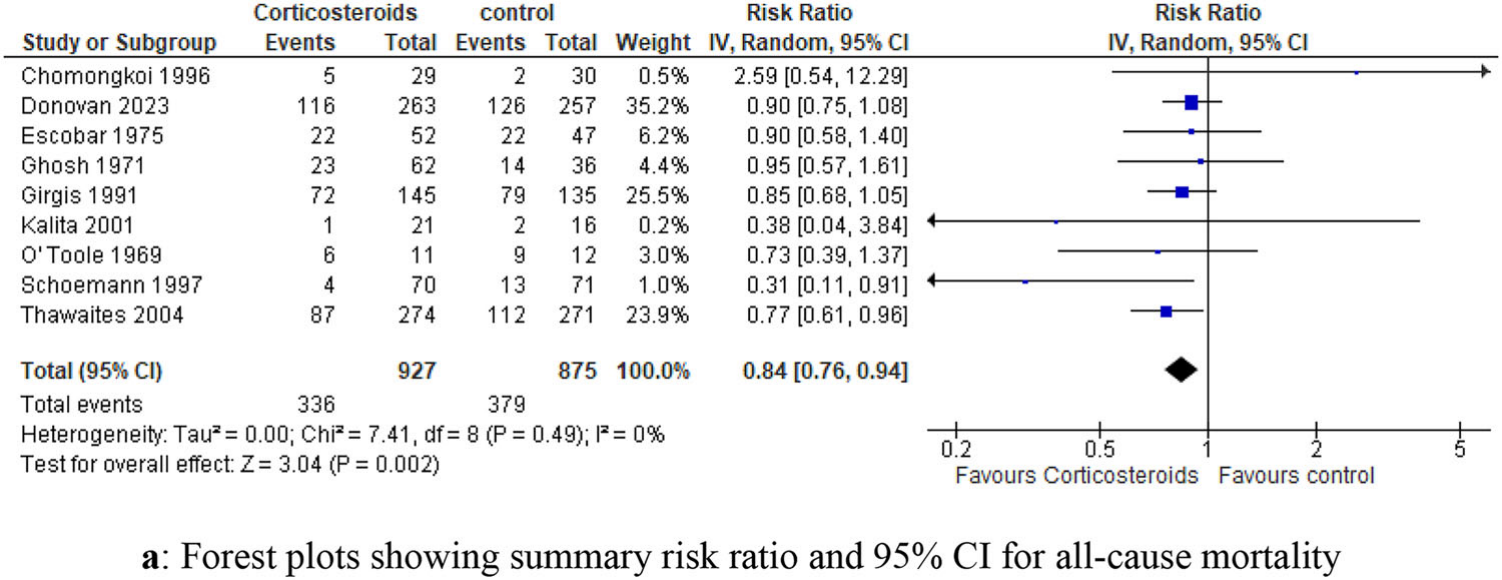
Forest plots showing summary risk ratio and 95% CI for all‐cause mortality.

**FIGURE 4b:**
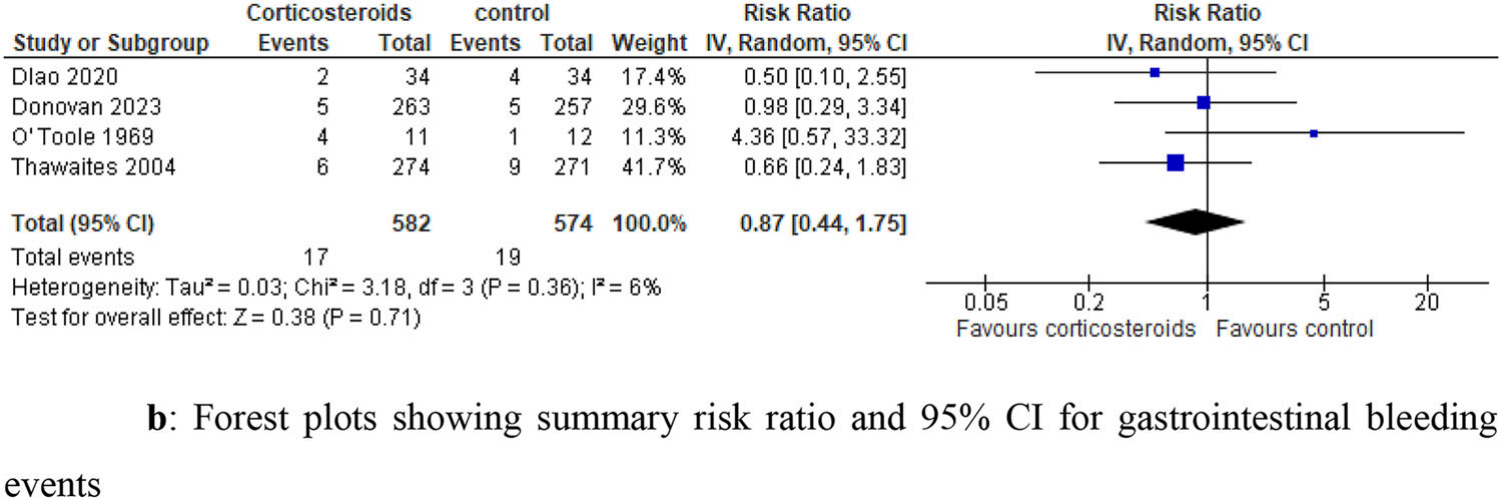
Forest plots showing summary risk ratio and 95% CI for gastrointestinal bleeding events.

**FIGURE 4c:**
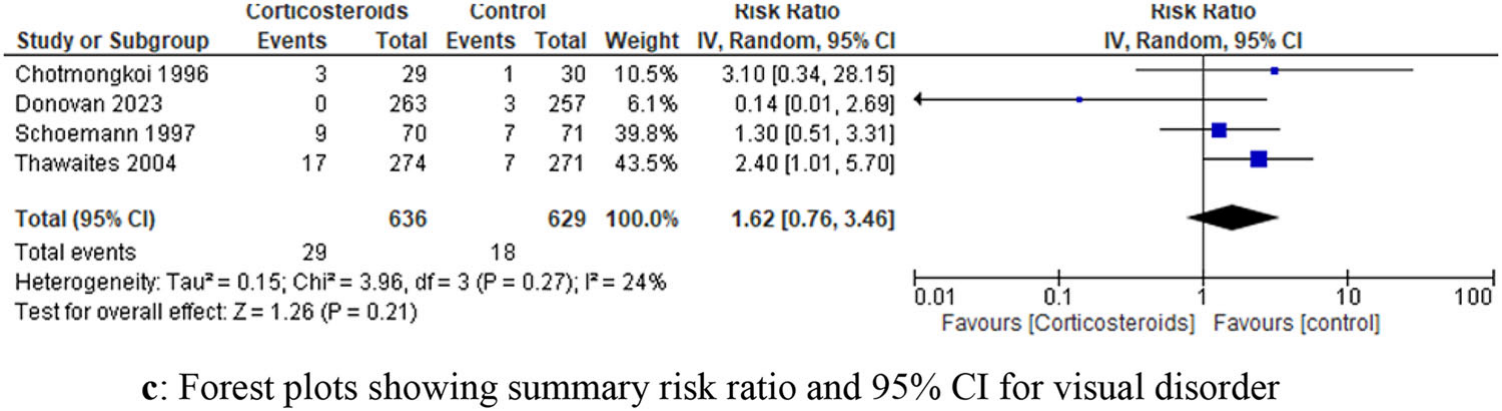
Forest plots showing summary risk ratio and 95% CI for visual disorder.

**FIGURE 4d:**
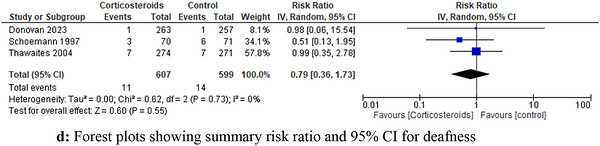
Forest plots showing summary risk ratio and 95% CI for deafness.

**FIGURE 4e:**
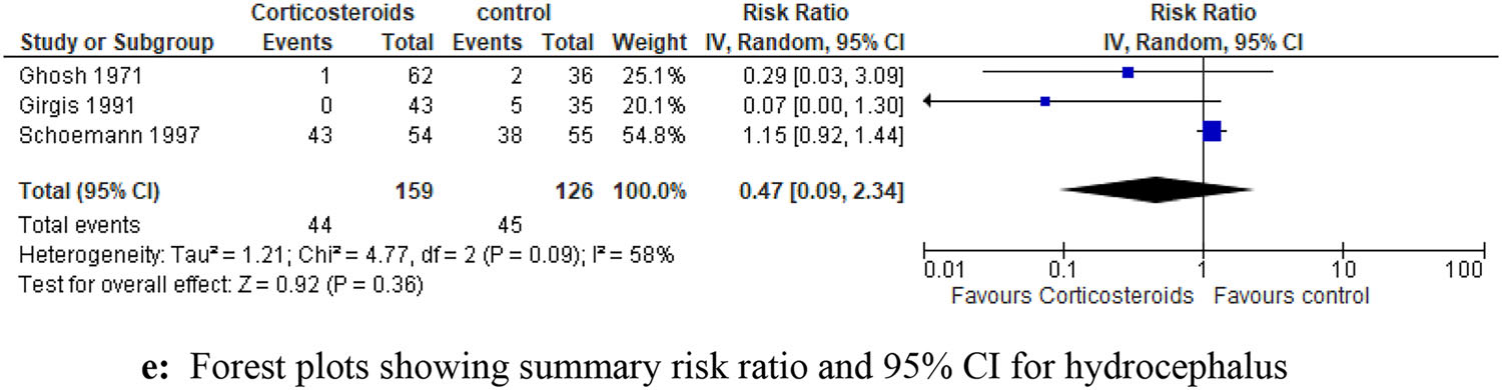
Forest plots showing summary risk ratio and 95% CI for hydrocephalus.

### 3.3 Clinical Outcomes

A meta‐analysis was carried out to clinically and quantitatively synthesize the clinical outcomes reported in the randomized controlled trials that were included. The first one was all‐cause mortality, or the total effect of adjunctive corticosteroid treatment on the survival of patients with tuberculous meningitis. The secondary outcomes were adverse events, which included gastrointestinal bleeding; visual disability (including reduced visual acuity, blindness, and blurred vision); deafness; and hydrocephalus and joint disorders. Each outcome had pooled risk ratios (RRs) and confidence intervals (CIs) of 95% random‐effects meta‐analysis (DerSimonian and Laird method) to consider inter‐study variability. All the analyses were performed on the intention‐to‐treat basis, and the heterogeneity was measured with the help of Higgins *I* ^2^ statistic to show the extent of variability across the studies with respect to each outcome.

### 3.4 Primary Outcome: (Figure 4)

#### 3.4.1 All‐Cause Mortality: (Figure 4a‐f)

Nine randomized controlled trials showed all‐cause mortality and included 1747 patients (around 905 in the corticosteroid group and 842 in the control group) (Chotmongkol et al. 1996; Donovan et al. 2023; Escobar et al. 1975; Ghosh et al. 1971; Girgis et al. 1991; Kalita and Misra 2001; O'Toole et al. 1969; Schoeman et al. 1997; Thwaites et al. 2004). The meta‐analysis showed that adjunctive corticosteroid interventions were associated with a significant reduction in increased risk of all‐cause mortality rate compared to using standard anti‐tuberculosis therapy alone (pooled RR = 0.84, 95% CI = 0.76–0.94, *p* = 0.002). This represents a statistically significant survival advantage with the use of corticosteroids. Heterogeneity between studies was low (*I* ^2^ = 0%), indicating that there is a unified direction of effect across all the included trials. There was no sign of publication bias that was identified, and this was established by the Egger regression test (*p* = 0.6017), which shows that the pooled estimate was reliable. Based on the GRADE assessment, the quality of evidence of this outcome was high, and the data are robust and consistent through the studies (Table 2).

**TABLE 2:** Grading of recommendations assessment, development, and evaluation (GRADE) summary of findings.

Outcome	Participants (studies)	Relative effect, RR (95% CI)	Risk of bias	Inconsistency	Indirectness	Imprecision	Publication bias	Certainty (GRADE)
**All‐cause mortality**	1747 (9)	0.84 (0.76 to 0.94)	Not serious	Not serious	Not serious	Not serious	Undetected	⨁⨁⨁⨁ **High**
**Gastrointestinal bleeding**	4 trials	0.87 (0.44 to 1.75)	Not serious	Not serious	Not serious	Serious^a^	Undetected	⨁⨁⨁◯ **Moderate**
**Visual disability**	4 trials	1.62 (0.76 to 3.46)	Not serious	Not serious	Not serious	Serious^a^	Undetected	⨁⨁⨁◯ **Moderate**
**Deafness**	3 trials	0.79 (0.36 to 1.73)	Not serious	Not serious	Not serious	Serious^a^	Undetected	⨁⨁⨁◯ **Moderate**
**Hydrocephalus**	3 trials	0.47 (0.09 to 2.34)	Serious^b^	Serious^c^	Not serious	Not serious	Undetected	⨁⨁◯◯ **Low**
**Joint disorders**	2 trials	0.63 (0.26 to 1.50)	Not serious	Not serious	Not serious	Serious^d^	Undetected	⨁⨁⨁◯ **Moderate**

***Certainty of evidence (GRADE)***: ⨁⨁⨁⨁ High, ⨁⨁⨁◯ Moderate, ⨁⨁◯◯ Low, ⨁◯◯◯ Very low.

Domain ratings use a uniform vocabulary (Not serious / Serious / Very serious). Publication bias was undetected for all outcomes by funnel‐plot inspection and Egger's regression test (p = 0.60).

**^a^** Downgraded one level for imprecision: the 95% confidence interval is wide and crosses the line of no effect (RR = 1.0), with a small number of events.

**^b^** *Downgraded one level for risk of bias: contributing trials had concerns in the randomization or outcome‐measurement domains on RoB 2 assessment*.

**^c^** *Downgraded one level for inconsistency: moderate between‐study heterogeneity (I^2^ = 58%)*.

**^d^** *Downgraded one level for imprecision: estimate derived from only two trials with few events and a wide confidence interval*.

**Patient or population**: patients with tuberculous meningitis. **Intervention**: adjunctive corticosteroids plus standard anti‐tuberculosis therapy. **Comparator**: placebo or standard anti‐tuberculosis therapy alone.

### 3.5 Secondary Outcomes

#### 3.5.1 Gastrointestinal Bleeding (Figure 4b)

Four trials were included in a meta‐analysis to determine the incidence of gastrointestinal bleeding between patients receiving adjunctive corticosteroid therapy and patients receiving standard anti‐tuberculosis regimens. The combined analysis did not demonstrate any statistically significant difference in the risk of gastrointestinal bleeding in the corticosteroid and control groups (RR = 0.87, 95% CI = 0.44–1.75, *p* = 0.71). This shows that the use of corticosteroids did not support or suppress the risk of gastrointestinal bleeding in patients with tuberculous meningitis. Inter‐study heterogeneity was low (*I* ^2^ = 6%), which indicates that the included studies were consistent. The certainty of the evidence for this outcome was rated as moderate according to the GRADE assessment, primarily due to imprecision in the pooled estimates (Table 2).

#### 3.5.2 Visual Disability: (Figure 4c)

Visual disability was reported by four studies (Chotmongkol et al. 1996; Donovan et al. 2023; Schoeman et al. 1997; Thwaites et al. 2004). Statistical analysis revealed that corticosteroids did not significantly increase the risk of visual defects in patients with tuberculous meningitis (RR = 1.62, CI = 0.76–3.46, *p* = 0.21), Figure 4(c). A low level of heterogeneity was observed (*I* ^2^ = 24%). The overall quality of evidence was moderate due to some concerns of imprecision in the GRADE domain (Table 2).

#### 3.5.3 Deafness: (Figure 4d)

Three studies reported deafness (Donovan et al. 2023; Schoeman et al. 1997; Thwaites et al. 2004). Our meta‐analysis revealed no statistically significant difference in risk of deafness between the corticosteroid and comparison arms (RR = 0.79, CI = 0.36–1.73, *p* = 0.55). The level of heterogeneity was quite low (*I* ^2^ = 0%), as shown in Figure 4(d). The overall quality of evidence was moderate due to some concerns of imprecision in the GRADE domain (Table 2).

#### 3.5.4 Hydrocephalus: (Figure 4e)

Hydrocephalus was reported by three studies (Diao et al. 2020; Donovan et al. 2023; Thwaites et al. 2004). Statistical analysis demonstrated no statistically significant difference between corticosteroids and the control arm in the risk of hydrocephalus (RR = 0.47, CI = 0.09–2.34, *p* = 0.36). The overall level of heterogeneity was found to be moderate (*I* ^2^ = 58%), Figure 4(e). The certainty of the evidence was found to be low due to some concerns of inconsistency and high risk of bias in the included studies (Table 2).

##### 3.5.4.1 Joint Disorders:(Figure 4f)

Joint disorders were reported by two studies (Kumarvelu et al. 1994; Schoeman et al. 1997). Our meta‐analysis provides evidence that there is no statistically significant difference in risk of joint disorders (RR = 0.63, CI = 0.26–1.50, *p* = 0.30) in patients receiving corticosteroids compared to those receiving standard AT regimen. The level of heterogeneity was quite low (*I* ^2^ = 0%), Figure 4(f). The certainty of the evidence was moderate due to some concern of imprecision in the GRADE domain (Table 2).

**FIGURE 4f:**
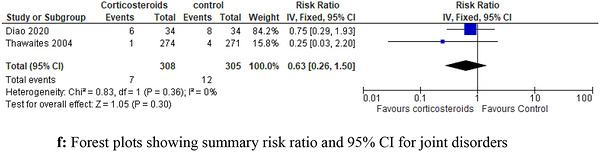
Forest plots showing summary risk ratio and 95% CI for joint disorders.

### 3.6 Subgroup Analysis of Primary Outcome:

#### 3.6.1 Upper‐Middle and Lower‐Middle Income Countries

The administration of corticosteroids significantly reduces the risk of all‐cause mortality in lower‐middle‐income countries (RR = 0.81, CI = 0.70–0.94, *p* = 0.005) but not in upper‐middle‐income countries (RR = 1.28, CI = 0.47–3.48, *p* = 0.65), Supplementary Figure S2a.

#### 3.6.2 Type of Steroids: Dexamethasone vs. Other Steroids

Dexamethasone significantly reduces all‐cause mortality (RR = 0.87, CI = 0.77–0.97, *p* = 0.02). Other steroids, however, do not affect the mortality rate (RR = 0.82, CI = 0.52–1.28, *p* = 0.38), (Supplementary Figure S2b).

#### 3.6.3 HIV‐Positive Patients

Corticosteroids did not reduce all‐cause mortality in HIV‐positive individuals (RR = 0.90, CI = 0.77–1.05, *p* = 0.18), (Supplementary Figure S2c).

#### 3.6.4 Meta‐Regression Analyses

A meta‐regression analysis was conducted to explore whether the duration of follow‐up across studies influenced the effect of corticosteroids on all‐cause mortality. The analysis was performed using R software (version 4.4.0) with the metafor and meta packages, applying a random‐effects restricted maximum likelihood (REML) model. Follow‐up duration (in months) was included as a continuous moderator variable. The regression coefficient (*β* = 0.3697, *p* = 0.5432) indicated that the length of follow‐up did not significantly modify the association between corticosteroid therapy and mortality in patients with tuberculous meningitis. Model diagnostics, residual heterogeneity (τ^2^), and regression plots confirmed the stability and non‐significance of this moderator effect. Full model output and the corresponding meta‐regression scatter plot are presented in Supplementary Figure S3.

### 3.7 Sensitivity Analysis

The funnel plot was visually inspected to assess potential publication bias and found no evidence of visual asymmetry. Egger's test also confirmed the absence of small‐study effects (*p* = 0.6017) (Supplementary Figure S1). Leave‐one‐out sensitivity analyses were conducted to evaluate the influence of high‐risk studies on the overall effect size. These analyses showed that the pooled estimate remained stable across iterations, and the exclusion of high‐risk studies and those with some concern with the bias did not substantially influence the results while heterogeneity remained low (*I* ^2^ = 0%) (Supplementary Figure S3a).

In the leave‐one‐out sensitivity analysis excluding the three high‐risk studies (Escobar 1975; Ghosh 1971; Kumarvelu 1997), the pooled risk ratio remained significant at RR = 0.84 (95% CI: 0.73–0.97, *p* = 0.02), with negligible heterogeneity (*I* ^2^ = 0%, *p* = 0.45), confirming that the primary result is not driven by these studies. In this restricted analysis, Donovan et al. (2023) contributed the highest weight (59.4%), followed by Thwaites et al. (2004) (40.2%). The minor widening of the confidence interval relative to the primary analysis (0.76–0.94) reflects the reduced sample size following exclusion of three trials, but the direction and significance of the mortality benefit are preserved.

Also, to further support the strength of our results, a sensitivity analysis was conducted by eliminating those studies that were published earlier before the year 2000. The respective forest plot showed that the general findings were not affected, as this illustrated that the inclusion of the older age trials did not have a significant effect on the aggregate results. Supplementary Figure S3b) The analyses confirm the consistency and reliability of the reported all‐cause mortality reduction with corticosteroid use in patients with meningitis caused by tuberculosis.

## 4 Discussion

### 4.1 Summary of Key Findings

This meta‐analysis and systematic review included 11 randomized and quasi‐randomized controlled trials (a total of 1917 patients with tuberculous meningitis (TBM)). Our findings demonstrate that adjunctive corticosteroid treatment, especially dexamethasone, is an effective intervention to lower all‐cause mortality in TBM patients. Mortality benefit was observed to be higher among HIV‐negative and low‐ and middle‐income countries. There was no meaningful elevation of risk of adverse events, such as gastrointestinal bleeding, visual impairment, hearing loss, hydrocephalus, or joint disorders. Subgroup analyses were used to verify the similarities in these results between various study populations, and sensitivity analyses demonstrated that the omission of older studies or high‐risk studies did not significantly change the results. The test conducted by Egger did not indicate the presence of any publication bias, and the heterogeneity was low (*I* ^2^ = 0%).

### 4.2 Comparison With Previous Studies

Our findings align with previous meta‐analyses regarding the safety and efficacy of corticosteroids in reducing mortality. A 2016 Cochrane systematic review and meta‐analysis, which included nine trials with 1337 participants (469 deaths), found that adjunctive corticosteroids reduce deaths by almost one quarter, although no significant effect was observed on long‐term neurological disability in HIV‐negative patients. However, uncertainty persists regarding the efficacy of corticosteroids in HIV‐positive individuals due to the limited data available for this subgroup (Prasad et al. 2016).

Another review, encompassing11 studies, demonstrated that combining dexamethasone with conventional antitubercular drug therapy can enhance the effectiveness of TBM treatment and lower the incidence of adverse reactions (Wang et al. 2022). While our findings support increased survival, we did not observe a statistically significant effect on adverse events. This discrepancy may be attributed to differences in sample size, predominance of included studies from a single region, variability in study quality, and potential biases influencing statistical outcomes.

Several clinical trials have also reported improved survival rates in patients treated with steroids, attributing this benefit to reduced meningeal inflammation and decreased spinal block (Ashby and Grant 1955; Kapur 1969). Conversely, an earlier study (1963) failed to observe any significant effect following 14 days of steroid therapy in 19 patients, compared to the control (Lepper and Spies 1963). Variations in study design, population characteristics, follow‐up duration, small sample sizes, and advancements in clinical management likely explain these conflicting results.

Interestingly, Donovan et al. concluded that corticosteroid therapy was not beneficial in TBM, which contrasts with our results demonstrating a significant mortality reduction (2023). Such deviation is not surprising, because in case of severe immunosuppression, the responsiveness to corticosteroids can be blunted, and patients can be vulnerable to secondary infections. In contrast, among HIV‐negative people, corticosteroids will probably prevent excessive inflammatory reactions, decreasing cerebral edema and intracranial pressure.

Like our study, a recent retrospective study found that patients with TBM receiving aspirin and corticosteroids as adjunctive therapy delineated reduced mortality compared to those receiving aspirin alone or no adjunctive therapy, even in the case of severe meningitis (Misra et al. 2018). However, this study's limited sample size and short 3‐month follow‐up period, along with introduced biases and variations in patient demographics, treatment protocols, and disease severity, may explain the lack of statistical significance in survival benefit.

A computed tomography‐based study suggested that corticosteroid treatment does not influence the burden of basal ganglia infarction (Schoeman et al. 1997). However, a serial MRI scan study demonstrated a 50% reduction in the burden of cerebral infarction among patients treated with dexamethasone compared to the control group (Thwaites et al. 2007). However, due to insufficient available data, no certain conclusion can be drawn regarding the efficacy of corticosteroid treatment in preventing cerebral infarction.

Subgroup analysis revealed that the mortality benefit was evident in HIV‐negative patients but not in those who were HIV‐positive. This aligns with findings from previous individual trials, such as Thwaites et al., and may be attributed to the immunosuppressed state in HIV‐positive individuals, which can increase vulnerability to opportunistic infections and limit the anti‐inflammatory benefits of corticosteroids (2004). Similarly, mortality benefits were observed in patients from low‐income and middle‐income countries but not in those from high‐income countries. One possible explanation is that high‐income settings offer more rapid diagnosis, superior neurocritical care, and timely initiation of anti‐tubercular therapy, which may reduce the relative impact of adjunctive corticosteroids. In contrast, patients in lower‐income settings may benefit more from the anti‐inflammatory properties of corticosteroids due to delays in diagnosis, limited access to intensive care, and greater disease severity (Bhatti et al. 2024; Raut et al. 2024; Shukla et al. 2023; Teo et al. 2021; Zarra et al. 2024).

### 4.3 Contextual Interpretation

The interaction of corticosteroids with the consequence of HIV status is different, and this fact highlights the role of immune competence in shaping the response to treatment. The persons who are HIV‐negative might have more benefit because of the preserved immunological control, whereas HIV‐positive patients might have a limited benefit because of the advanced immunodeficiency. In the same way, the observed larger mortality decreases in low‐ and middle‐income environments can be due to the severity of underlying disease, later onset and subsequent diagnosis, and reduced availability to neurocritical care, which increases the relative benefits of anti‐inflammatory treatment.

Although some older studies, like that of Lepper and spies et al. (Lepper and Spies 1963) have found no such effect of corticosteroid, these trials were done before standardized anti‐tuberculosis regimens and modern neuroimaging, rendering their use in modern practice rather limited.

### 4.4 Strengths of the Study

The meta‐analysis has a number of strengths. It is one of the most thorough reviews of randomized evidence on corticosteroid use in TBM that has been conducted to date, exemplifying recent RCTs issued until May 2025. The transparency and reproducibility were determined because the methodology followed PRISMA 2020 and Cochrane guidelines. Clinical trials were included only, which reduced the confounding factor of the observational designs. Such high‐tech methods as subgroup and meta‐regression analyses were used to examine the effect modifiers and strengthen the results. Moreover, the pooled estimates were stable and reliable, as was confirmed by publication bias and sensitivity analysis.

### 4.5 Limitations

Regardless of these advantages, there are some weaknesses that should be mentioned. The studies included had heterogeneity in terms of corticosteroid type, dose, and duration, which also played a role in clinical heterogeneity, which could not be accounted for statistically. The interpretability of subgroup results was restricted because of incomplete reporting of key variables, in particular, the HIV status and baseline severity of the disease. The limited number of possible RCTs and poor representation of children and HIV‐positive populations limit the external validity. Earlier studies were not standardized in their outcome measures and up‐to‐date imaging, and this could have underreported neurological outcomes. Because eligibility was restricted to studies available in English, relevant trials published in other languages may have been missed, introducing potential language bias.

### 4.6 Future Directions and Implications

The future randomized controlled trials must prioritize certain at‐risk subgroups like HIV‐positive individuals and children, where the existing information is sparse. Outcome measurement (particularly neurological sequelae and quality of life) will have to be standardized to enhance comparability. A combination of biomarkers and neuroimaging parameters can also help to clarify the mechanistic findings of corticosteroids on cerebral inflammation. Clinically, our results justify the use of the adjunctive corticosteroids, especially dexamethasone, in the treatment of TBM in HIV‐negative patients, especially in low‐resource areas. This should, however, be done with caution by policymakers and clinicians among HIV‐positive populations until more specific evidence is available.

## 5 Conclusion

This systematic review and meta‐analysis demonstrates that adjunctive corticosteroid therapy, especially dexamethasone, is associated with a significant reduction in all‐cause mortality in people with TBM, especially HIV‐negative individuals and those in low‐ and middle‐income countries. Corticosteroids provide a definite survival benefit without raising the risk of significant adverse events, including gastrointestinal bleeding, hydrocephalus, visual or auditory impairment, and joint disorders. These findings support the use of corticosteroids as a complement to conventional anti‐tuberculosis treatment in enhancing the clinical outcomes of patients with TBM.

The lack of proven advantage to the HIV‐positive population, however, indicates the necessity of individual approaches to treatment and additional targeted studies. Large, high‐quality randomized controlled trials in future investigations are mandatory in order to confirm these results in different populations, to optimize corticosteroid doses and time, and to measure long‐term neurological consequences. Taken together, these data reinforce the existing treatment guidelines of the world and justify the further application of corticosteroids as one of the adjunctive therapies to treat TBM, but with strict caution in immunocompromised patients.

## Author Contributions

**Ayesha Younas**: conceptualization, investigation, writing – original draft, methodology. **Muhammad Sameer Almas**: data curation. **Rizwana Noor**: software. **Muhammad Talha Shaukat**: methodology. **Norma Nicole Gamarra Valverde**: validation. **Murtaja Satea**: writing – review and editing. **Rosheen Jamil**: formal analysis. **Kashif Qureshi**: writing – review and editing, visualization. **Maria Qadri**: visualization, validation. **Kivanc Yangi**: writing – review and editing. **Irra Tariq**: investigation. **Bipin Chaurasia**: writing – review and editing, visualization, validation, supervision. **Aisha Ijlal**: validation. **Mahwash Siddiqi**: investigation. **Mifrah Rahat Khan Sherwani**: supervision, writing – review and editing.

## AI Usage Declaration

Artificial intelligence (AI) tools were not used in the collection, extraction, statistical analysis, or interpretation of data in this study. AI‐assisted writing tools (large language models) were used solely to assist with grammar and language editing of the manuscript draft. All scientific content, conclusions, and critical revisions were performed by the authors, who take full responsibility for the integrity of the work.

## Funding

The authors have nothing to report.

## Conflicts of Interest

The authors declare no conflicts of interest.

## Figure Copyright Statement

All were generated de novo by the authors using RevMan 5.4.1, R version 4.4.0, and Comprehensive Meta‐Analysis software for this study. The central illustration (Figure 1) was created by the authors. No figures were reproduced or adapted from external copyrighted sources.

## Supporting information



Supplementary Figure S1 Funnel plots for all‐cause mortality.
Supplementary Figure S2(a) Subgroup analysis on upper‐middle and lower‐middle income countries.
Supplementary Figure S2(b) Subgroup analysis on the type of steroid.
Supplementary Figure S2(c) Subgroup analysis on HIV+ individuals.
Supplementary Figure S3(a) Sensitivity analysis on high‐risk studies on all‐cause mortality.
Supplementary Figure S3(b) Sensitivity analysis excluding old trials from all‐cause mortality.
Supplementary Figure S4 Meta‐regression scatter plot.

## Data Availability

The data that support the findings of this study are available from the corresponding author upon reasonable request.
